# Soft robotics for infrastructure protection

**DOI:** 10.3389/frobt.2022.1026891

**Published:** 2022-11-10

**Authors:** Edoardo Milana

**Affiliations:** Institute for the Protection of Terrestrial Infrastructures, German Aerospace Center (DLR), Sankt Augustin, Germany

**Keywords:** soft robotics, infrastructures protection, pipelines inspection, rubble search and rescue, soft aerial manipulation, safety and security

## Abstract

The paradigm change introduced by soft robotics is going to dramatically push forward the abilities of autonomous systems in the next future, enabling their applications in extremely challenging scenarios. The ability of soft robots to safely interact and adapt to the surroundings is key to operate in unstructured environments, where the autonomous agent has little or no knowledge about the world around it. A similar context occurs when critical infrastructures face threats or disruptions, for examples due to natural disasters or external attacks (physical or cyber). In this case, autonomous systems may be employed to respond to such emergencies and have to be able to deal with unforeseen physical conditions and uncertainties, where the mechanical interaction with the environment is not only inevitable but also desirable to successfully perform their tasks. In this perspective, I discuss applications of soft robots for the protection of infrastructures, including recent advances in pipelines inspection, rubble search and rescue, and soft aerial manipulation, and promising perspectives on operations in radioactive environments, underwater monitoring and space exploration.

## 1 Introduction

The ability of securing critical infrastructures in case of sudden disruptions is paramount to ensure the supply of goods and services that guarantee the stability of our societies (Lichte et al., 2022). In this context, advanced autonomous systems (mobile robots, drones, sensor networks) are being employed not only for monitoring the correct functioning of infrastructures but also to intervene in case of an emergency, either man-made or caused by natural disasters, leading to the formation of the scientific community of Safety, Security and Rescue Robotics (Murphy and Kleiner, 2013). The use of autonomous systems can be explained by several reasons, including prompt and frequent deployment, reduced operational costs and full digitisation of the tasks. Nevertheless, the most compelling reason is surely the need of protecting human lives, minimizing the safety risks that operators and civil protection practitioners undergo during maintenance and emergency responses.

The robots that populate our factories and revolutionized manufacturing processes consist of clever assemblies of rigid links and electric motors. They are typically made of steel or other stiff materials, which make them heavy and sturdy, very dangerous in case of collisions with humans operators. This is the reason why we are used to see industrial robots relegated into cages or in specific areas with limited access of personnel. Moreover, for the sake of performing specific and repetitive tasks, robots have been made even stiffer by implementing controllers to improve accuracy and repeatability of the motion and avoid any unintended contact (Della Santina et al., 2017). While this approach revealed to be extremely successful in the industrial context, it appears to be detrimental for robots that are intended to operate outside the factories, in scenarios where tasks are uncertain and unique and the presence of humans is ubiquitous. We refer to this last type of scenarios as unstructured environments as it could be a disrupted infrastructure, whereas assembly lines and warehouses are typically structured environments.

With these premises, soft robotics aims at introducing elasticity in the robot body, decreasing the overall stiffness, while embracing and harnessing mechanical interaction with the environment (Albu-Schaffer et al., 2008; Rus and Tolley, 2015). The elasticity can be localized in the joints (articulated soft robots) by adding springs in the actuators or can be distributed in the whole robot body (continuum soft robots) by using soft materials, such as elastomers, gels and biohydrid materials (Della Santina et al., 2020). Continuum soft robotics has recently attracted a lot of attention due to its multidisciplinary nature, combining expertise in materials science and solid mechanics. Soft robotics is anticipated to foster the use of autonomous systems in fields where human-robot interaction is preeminent, such as minimally invasive surgery, rehabilitation devices, elderly care, virtual reality, housekeeping etc (Majidi, 2014; Cianchetti et al., 2018).

In this paper I share thoughts on how soft robotics can be a game-changer regarding the deployment of autonomous systems for infrastructures protection. In such extremely unstructured environments, where there are multiple uncertainties and on-going risks, providing autonomous systems with extremely sophisticated artificial intelligence algorithms, which drastically improve their perception and planning abilities, is certainly fundamental, but it would not be enough to guarantee human-like performances as long as the mechanical behaviour of such systems stays poor. With a poor mechanical behaviour it is meant a system that does not make an intelligent use of the physical properties of its body and, therefore, not only cannot interact and adapt to the environment either passively or actively, but also cannot store and release elastic energy to increase its energy efficiency while performing tasks. Those concepts are well known under the umbrella term of embodied intelligence (Pfeifer and Bongard, 2006), a paradigm in robotics, neuroscience, psychology and philosophy that stresses on the fact that the intelligent abilities of an agent do not depend only on the brain but also on the body, *via* the physical interaction with the environment.

**FIGURE 1:**
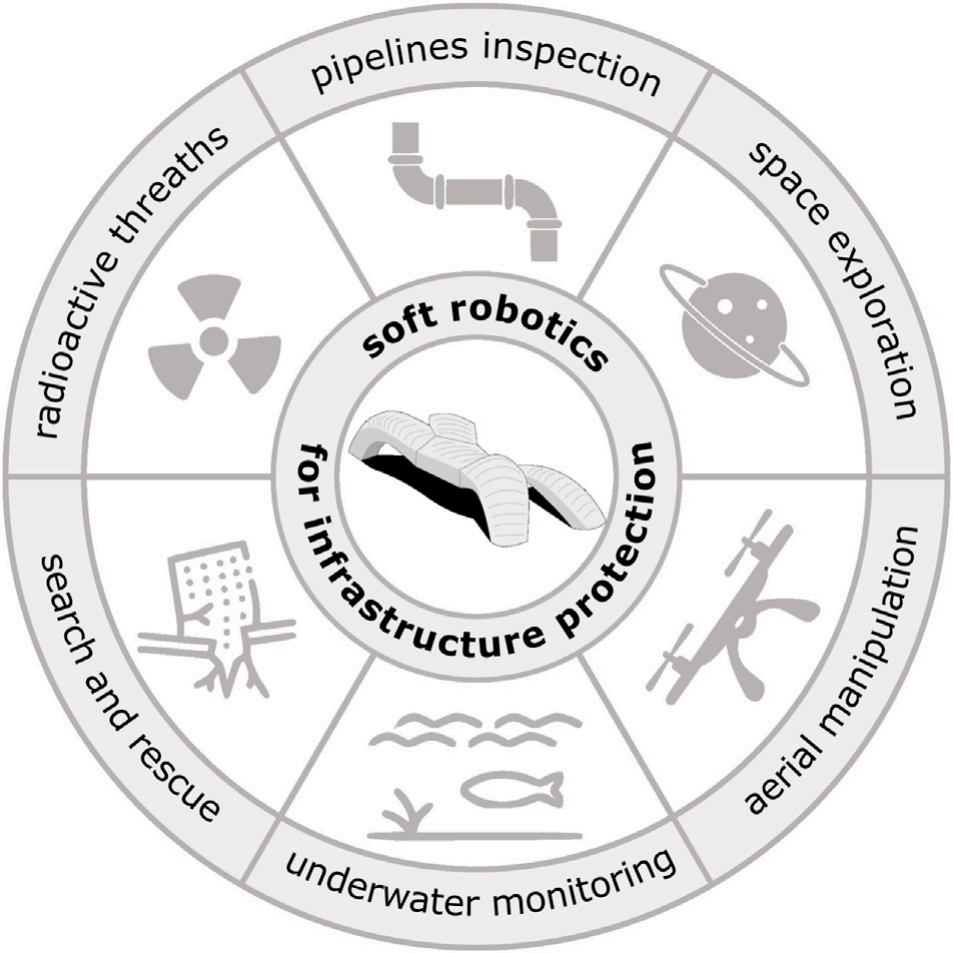
Schematic overview of possible areas of application of soft robotics in the context of the protection of critical infrastructures, such as inspection and monitoring of pipelines, rubble search and rescue, soft aerial manipulation, underwater monitoring and exploration, operations in radioactive environments and space exploration.

In the next sections, I report examples from the literature that show promising applications of soft robotics for the protection of infrastructures as summarized in Figure 1. Finally I discuss current limitations and give an outlook on future developments.

## 2 Soft robotics for infrastructure protection

### 2.1 Pipelines inspection

Pipelines are a fundamental component of terrestrial infrastructures and their correct operations are vital to the stability of our societies, considering freshwater supply and sewage. Pipelines inspection can therefore prevent failure events that may lead to a critical disruption of the infrastructure. Robotic systems are a preferable solution over human maintenance, for cost and safety reasons, since it is not required to shut down and empty the pipe (Adams et al., 2018). However, rigid robots are limited in terms of flexibility needed to navigate pipes of different dimensions, which is the reason soft robots are anticipated to outperform them.

**FIGURE 2:**
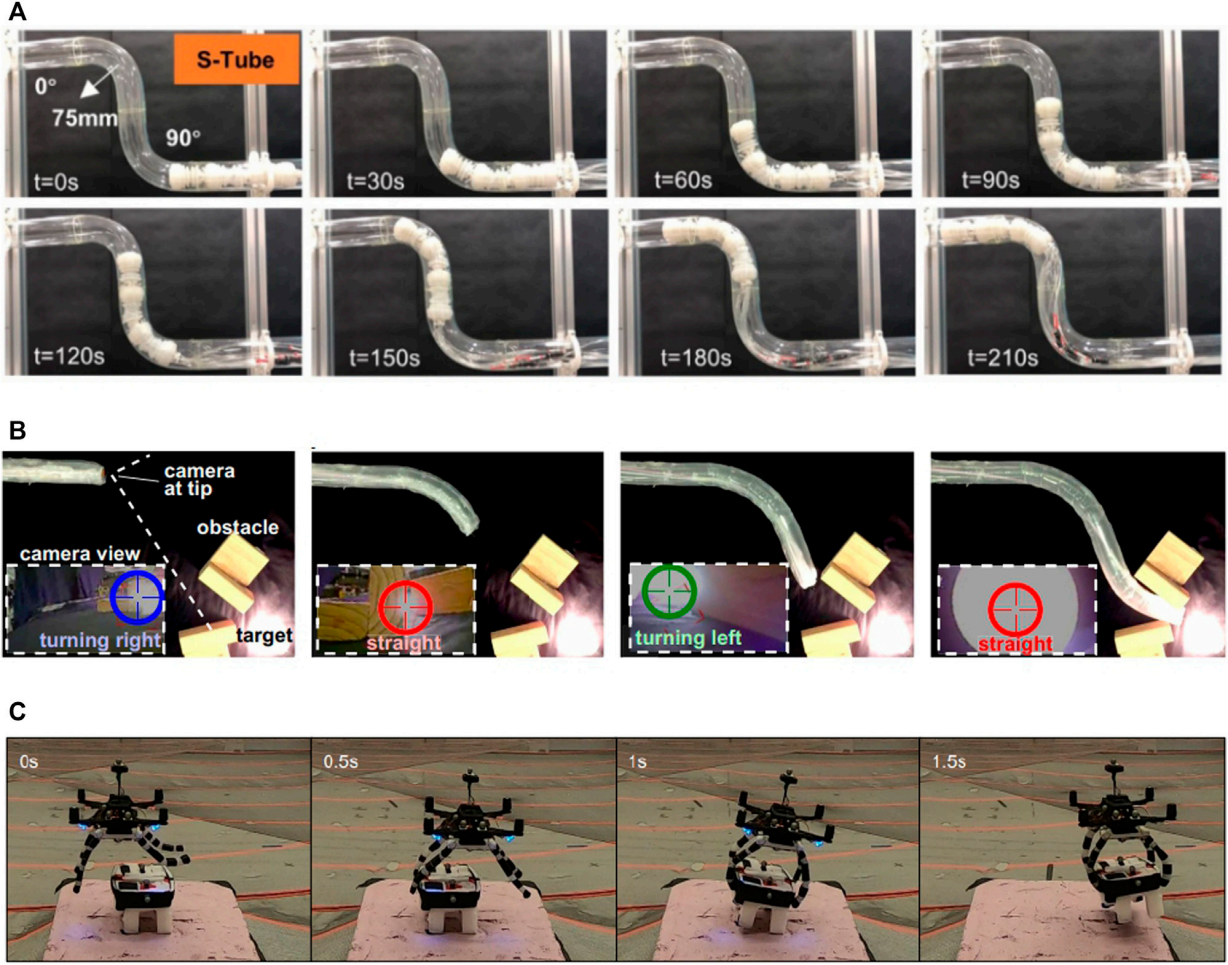
Examples of soft robotic systems performing tasks useful for infrastructure protection. **(A)** Soft crawler navigating an S-shaped pipe [Figure adapted from Liu et al. (2022), (CC BY 4.0)]. **(B)** Inspection skills of a vine robot [Figure adapted from Hawkes et al. (2017), © [2017] AAAS. Reprinted with permission]. **(C)** Aerial dynamic grasping using a soft gripper [Figure adapted from Fishman et al. (2021), © [2021] IEEE. Reprinted with permission].

Most soft robots designed for pipes inspections are inspired by the movement of inchworms, and perform a peristaltic crawling locomotion across the tubular shape of the pipe. Those robots typically use flexible components at their extremities to anchor to the inner surface of the tube, and extending actuators distributed along the body to move forward. The anchors can consist of passive elements like rubber membranes with embedded grooves for differential friction (Zhang Z. et al., 2019) or active elements, such as radially expanding inflatable actuators, that can be made of fabrics (Adams et al., 2018) or rubber (Zhang B. et al., 2019, 2018; Liu et al., 2022). Additional extending actuators can be added to the structure of such robots to give them the ability to turn in presence of a pipe split (Zhang et al., 2019a,b). Liu et al. (Liu et al., 2022) demonstrated some functional characteristics for pipes inspections (Figure 2A), such as following the pipe path regardless the direction of gravity, carrying weights, moving in dry, wet and oily pipes, removing objects from the pipes and moving underwater. The soft robot of Zhang et al. (Zhang et al., 2018) was succesfully tested in pipes with varying diameters. Other authors were able to embed a camera in the robot (Zhang et al., 2019b,a), actually providing the inspection ability.

### 2.2 Rubble search and rescue

Collapses of buildings due to explosions (bomb attacks or accidental gas leakages) or natural disasters (earthquakes or flooding) may unfortunately result in people trapped under the rubble. In this scenario, a robotic system deployed for search and rescue has to be able to navigate narrow and cluttered environments to locate people and establish a communication, undergoing inevitable interaction between the robot and the rubble, which may cause further collapses. Soft robots are ideal candidates not only due to the low forces exerted during those interactions, but also due to their ability to harness mechanical contact as feedback for navigation.

A class of soft robots that have a great potential for this task are growing robots. Growing robots (growbots), as one may deduce, consists of robotic systems that expand their body from the tip, inspired by the movements of plants’ roots. Contrary to mobile soft robot, growing robot have the advantage of a fixed base that can house the energy supply and the control systems, avoiding the technical challenge of integrating such components on board of the soft robot body. The growing tip has the directional control, enabling steering, while the “grown body” maintains its deformed shape. Growing can be achieved through continuous 3D printing (Sadeghi et al., 2017) or by inflation of inverted thin-wall tubes (so-called vine robots), either single tube (Hawkes et al., 2017; der Maur et al., 2021) or multiple small tubes (Tsukagoshi et al., 2011). In 3D printing growbots, steering is achieved through a differential material deposition along the cross-section, resulting in the formation of a curvature. Vine robots (Figure 2B), instead, can steer through an asymmetric lengthening of the inflated tip. In both systems the tip acts as sensors’ carrier to enable monitoring and inspections tasks, using cameras (Tsukagoshi et al., 2011; Hawkes et al., 2017), IMUs, speaker and microphone for communicating with possible trapped persons (der Maur et al., 2021), environmental sensors (Sadeghi et al., 2017).

Regarding tests in realistic environments, RoBoa, a vine robot developed at ETH Zurich (der Maur et al., 2021), has been successfully deployed in a debris site used for training civil security practitioners. The robot was teleoperated to navigate through rubble and locate a trapped person 10 m far from the growing starting point, showing the potential of such soft robotic systems.

### 2.3 Soft aerial manipulation

In critical situations it is often not sufficient to deploy autonomous systems that can only perceive the environment, but it is necessary that they are provided with the ability of performing an action, which may be needed to restore the security of an infrastructure. In this context, aerial robots are widely employed due to their fast motion and independence on the ground conditions, and, therefore, implementing a gripper on an aerial robot is a promising approach to equip autonomous systems with grasping capabilities. A thorough review of aerial manipulation is reported in (Bonyan Khamseh et al., 2018).

Recently, within the paradigm of soft robotics, many new designs of soft hands and soft grippers have been proposed, which harness the compliance of the fingers to grasp objects of different shapes and materials with a reduced amount of computation in the control system (Shintake et al., 2018). Accordingly, such soft grippers are being implemented on aerial robots to enable universal and dynamic grasping. Underactuated tendon-driven grippers have been the earliest implementation of soft aerial manipulation, proving its feasibility with retrieving unstructured objects (Ghadiok et al., 2011; Pounds et al., 2011). Soft bag actuators with variable restriction mechanism to control the bending motion have been used in an aerial manipulator that can push doors and twist knobs (Tsukagoshi et al., 2015). Four completely soft tendon-actuated fingers have been attached to a quadrotor to grasp objects of unspecified shapes during fast movements (Figure 2C), inspired by the grasping capabilities of biological systems (Fishman et al., 2021). Similarly, RAPTOR (Appius et al., 2022) is a soft aerial manipulator equipped with a customized Fin Ray^®^ gripper (Festo and KG, 2018) to also perform fast aerial grasping, reporting grasping velocities up to 1 m/s.

### 2.4 Further potential applications

The three applications reported in the previous sections are currently the most advanced in the context of soft robotics for infrastructure protection, based on the success of the proof of concepts reported by the authors. However, there are also perspectives about the advantage of adopting soft robotic technology in other aspects of critical infrastructures, such as autonomous operations in radioactive environments (Vitanov et al., 2021) and even space exploration (Zhang et al., 2022). Yirmibesoglu et al. (Yirmibeşoğlu et al., 2019) studied the impact of gamma radiation on the mechanical properties of polydimethylsiloxane (PDMS), the most common silicone rubber used for soft robotic fabrication. They found a degradation of the mechanical properties with increasing exposure to radiation, but with minimal effect up to 20 kGy, inducing the authors to conclude that there is a great promise for such application. Regarding space exploration, Zhang et al. (Zhang et al., 2022) note that soft robots can potentially solve the technological challenges that occur in space, related to high-vacuum, microgravity, radiations and extreme temperatures, due to their advanced materials and structural flexibility.

An additional thematic area that is key for the protection of infrastructures is underwater soft robotics. Maritime infrastructures are as critical and vital as terrestrial ones, considering the strategic importance of underwater pipelines, offshore wind power and oil and gas, and naval monitoring. Moreover, fishes, octopuses and other aquatic organisms are a preeminent bioinspired paradigm for soft robots due to their flexible bodies and their swimming technique that can be mimicked by soft actuators (Cianchetti et al., 2015; Katzschmann et al., 2018). Indeed, bioinspired soft robots are foreseen as future technology for unmanned underwater vehicles (UAVs) thanks to the capability to adapt to the maritime environment (Youssef et al., 2022) and withstand extreme conditions as high pressures and hydrodynamic perturbation (Aracri et al., 2021).

## 3 Current limitations and perspectives

Current limitations and problems are common to any type of soft robots and reflect some fundamental challenges that need to be addressed to push the technology further.

Soft robots are still largely tethered to their energy supplies and control units, posing a serious limit to the operational space. As discussed earlier, this problem does not concern growing robots with a fixed base, but is particularly pressing for soft crawlers that inspect pipelines, where the robot is supposed to autonomously move for tens of meters. At this current stage of technology, softness is mainly confined to the actuators and the structural elements of the robot body, whereas other components (pumps, batteries, electronics, cameras) are still made of rigid materials, compromising the overall flexibility and increasing the weight of the system. Therefore, at the laboratory level, such robots are tethered to external pumps and the focus is on the locomotion performances. However, recent research works are tackling those issues, elaborating designs and manufacturing of soft pumps (Cacucciolo et al., 2019; Tang et al., 2021), soft computing systems (Garrad et al., 2019; Preston et al., 2019) and flexible batteries (Mukherjee et al., 2021). Alternatively, a reduction of the number of tethers can be achieved by mechanically programming simple control tasks (e.g. a pattern of actuation sequences) in the hardware of the system, by harnessing the nonlinear response of soft actuators (Gorissen et al., 2019; Milana et al., 2022) or soft valves (Rothemund et al., 2018; van Laake et al., 2022).

Soft manipulation has proofed universal grasping performances and its success relies on the fact that the compliant fingers passively conform to the objects thanks to the mechanical deformation. This allows the control system to be reduced to a switch between a “grasp” and “no-grasp” state and it typically requires no sensor feedback to successfully operate. However, the absence of the “sense of touch” limits the understanding of the grasping failure, as the system cannot determine whether the force applied is not sufficient or the gripper is wrongly positioned. The soft grippers implemented in the aerial manipulators reported in the previous section do not have this sense of touch, while it would be extremely beneficial, for example during dynamic grasping, to compensate for extra inertial forces generated by the grasped objects. Sensorizing soft grippers is not straightforward as the vast majority of commercially available force and strain sensors are made of rigid materials, limiting the structural compliance as mentioned also in the previous paragraph. Innovative solutions consist of highly stretchable strain sensors that can be directly embedded in the soft fingers, for example using 3D printed ionic conductive gels (Truby et al., 2018) or stretchable optical waveguides (Zhao et al., 2016). Other sensing technologies applied in soft grippers are reported by Shintake et al. in their literature review (Shintake et al., 2018). Another promising alternative is the GelSight technology, which consists of a combination of computer vision and soft materials for tactile reconstruction (Johnson and Adelson, 2009). Recently, Liu and Adelson presented a Fin Ray^®^ gripper with integrated a GelSight sensor to sense object orientation and deformation fields while grasping an object, performing reorientation and placement of a delicate object such as a wine glass (Liu and Adelson, 2022).

Most of the soft robotic systems reported in the previous section have some active control rules to make them operate fully autonomously. Soft aerial manipulators have implemented trajectory optimization and adaptive control, pipeline crawlers passively follow the tube shape due to their bodily compliance and growing robot can actively steer following visual or other sensory feedback. However, such control schemes, which are successfully implemented in lab environments, may be inadequate or insufficient in real world scenarios. In fact, the growing robot RoBoa has been teleoperated when tested in a realistic rubble search and rescue task. Complete autonomous operations in complex unstructured scenarios still requires research and development efforts from both the software and hardware side, and a prompter use of soft robotic systems for the protection of infrastructures can be the remote control from a civil security practitioner or other trained personnel. This would make it possible to deploy a soft robotic system without risking human lives and at the same time involving humans in the planning and decision of the robot actions.

A compelling problem that soft robots share with their rigid counterparts is energy consumption, which severely limits the deployment time of such autonomous system. This is particularly important in fields operations where the tasks are not clearly defined (e.g. response to emergencies) and therefore several hours of autonomy should be guaranteed. Legged robots like Boston Dynamics Spot are starting to be used for infrastructure protection due to their ability of walking on uneven terrains and climbing stairs, but their operational autonomy last maximum 90 min^1^, if the batteries are new and not powering any additional payload. Legged robots as well as soft robots are generally inspired by biological systems, whereas the latter can obviously “operate” much longer. In fact, biological systems make use of distributed energy sources and their bodily components are strictly interconnected and multifunctional, while current robots are designed as assemblies of separated building blocks (structure, actuators, sensors, control and power units), making them less efficient at using energy to perform tasks (Aubin et al., 2022). In this direction, recent trends in soft robotics show efforts in creating autonomous systems that exhibit some degrees of hardware multifunctionality (Aubin et al., 2022).

Mechanical properties aside, another advantage of using soft robots is their low cost and simplicity, due to the use of inexpensive silicone rubbers and the low amount of components to assembly. This makes it economically viable to use a large number of operating devices and, depending on the application, disposable soft robots as well.

## 4 Conclusion

Intelligent autonomous systems are an asset for infrastructure protection, from monitoring correct operations to emergency response. Recent developments of soft robotics are pushing the use of innovative autonomous systems for specific tasks where entirely rigid robots may fail or underperform, such as pipe inspection, rubble search and rescue, and soft aerial manipulation. Further, there are promising perspectives of soft robots being deployed for operations in radioactive environments, underwater and even in space. We discussed the current technological limitations in such applications and highlighted new promising solutions to overcome them, spanning between the design of soft basic computers, sensors and batteries and the augmented multifunctionality of the robot hardware. In perspective, the embodied intelligence approach, inspired by the biological systems that we aim at mimicking, will be paramount to build the next generation of robots with augmented duration of autonomy, to be deployed for infrastructure protection and other challenging unstructured environments.

## Data Availability

The original contributions presented in the study are included in the article/Supplementary Material, further inquiries can be directed to the corresponding author.
